# Engaging transgender women in HIV research in South Africa

**DOI:** 10.1186/s12889-023-15977-1

**Published:** 2023-05-29

**Authors:** L. Leigh Ann van der Merwe, Allanise Cloete, Helen Savva, Donald Skinner, Gita November, Zsa-Zsa Fisher

**Affiliations:** 1 Social, Health, and Empowerment (S.H.E) Feminist Collective of Transgender Women of Africa, East London, South Africa; 2 Public Health, Societies and Belonging (PHSB) the Human Sciences Research Council (HSRC), Cape Town, South Africa; 3 Prevention Branch, U.S. Centers for Disease Control and Prevention (CDC), Pretoria, South Africa; 4 School of Public Health, Stellenbosch University, Stellenbosch, South Africa; 5 Trans TEC SA, Cape Town, South Africa; 6 Trans Power Care Centre, Johannesburg, South Africa

**Keywords:** Transgender women, South Africa, Community-based participatory research, HIV research, Community engagement, Marginalized communities

## Abstract

The *Botshelo Ba Trans* study was the first HIV bio-behavioral survey conducted with transgender women in South Africa. Engaging research with marginalized communities requires clear points of entry, reference points for understanding the internal culture, and establishing trust and understanding. The community-based participatory research approach guided the development and implementation of this study. We conducted a rapid qualitative and pre-surveillance formative assessment between August 2017 to January 2018 and a bio-behavioral survey between July 2018 and March 2019. At the start, a Steering Committee, comprising primarily of transgender women, was established and subsequently provided substantial input into the mixed methods study conducted in Buffalo City, Cape Town, and Johannesburg. Key to the study's success was building trust and establishing ownership of the survey by transgender women recognized as expert knowledge holders. Thus, a community-based participatory research-informed approach enhanced the validity of the data and ensured that we addressed relevant issues.

## Background

Transgender women (TGW) have a disproportionately high burden of Human Immunodeficiency Virus (HIV) in several regions, including developed, middle-income, and developing countries [1–9]. TGW are at elevated risk for HIV infection, with a pooled prevalence of 25% across eight southern African countries, excluding South Africa [10].

In a qualitative study conducted in South Africa, TGW spoke in interviews about the challenges that they face around HIV and, more generally, in their lives, leading to high rates of infection [11, 12]. These include social rejection by family, persistent harassment, and victimization for their gender non-conformity during childhood, which sometimes leads to violence, living in extreme poverty, homelessness, and barriers to accessing healthcare services because of transphobia [11–13]. Experiencing various forms of marginalization permeates throughout the life course of TGW. These social oppressions are even more pronounced for 'black' and 'colored' TGW [14, 15]. According to Sevelius [15], HIV risk for TGW is embedded in multiple co-occurring public health problems, including poverty, violence, victimization, and discrimination, impacting TGW's mental well-being.

Often TGW are discriminated against by the social structures that should provide social and legal protections even within contexts where legislation protects the rights of TGW [16–18]. Given the persistent stigmatization experienced by TGW, mistrust and skepticism towards various sectors of society becomes an automatic response for many in this community. Mistrust and suspicion are not only informed by the stigmatization experienced by TGW in healthcare and other formal institutions, social rejection from structures, such as those expected from kinship ties, push TGW to the social and economic margins of society. For instance, stigma and mistrust towards the scientific community were reported by TGW as barriers to participation in a study on factors influencing the participation of TGW in HIV vaccine trials [19, 20]. In another study with TGW living with HIV, investigators found that TGW had concerns about being exploited, dehumanized, and judged [21].

Moreover, for TGW, participation in research studies presents an added financial burden. Often TGW are expected to travel to research sites [22]. In addition to the added financial burden, there is a suspicion amongst TGW that they might not receive monetary compensation for taking part in research [23]. Given this social context, engaging TGW in health research poses a challenge not only for estimating HIV prevalence but also for defining the HIV care continuum for transgender women, which can inform programmatic efforts needed to curb new HIV infections or improve treatment services [24].

### Community-based participatory research

Against this background, any research conducted with TGW should be grounded in social justice and executed within a community-based participatory framework. Research within a social justice paradigm promotes inclusivity and acknowledgment of marginalized groups' voices, experiences, and practices [25] and, importantly, upholds the principles of community-based participatory research (CBPR).

CBPR "builds bridges between scientists and communities by involving community participants and researchers" [26] from the conceptual/protocol development phase to the analysis and dissemination of study findings. Thus, a CBPR approach presupposes a co-learning process involving local community capacity building. It is an empowering process whereby participants can increase control over their lives and is a balance between research and action [27]. Trust is critical in this approach and can be harnessed through collaboration with the research participants [28]. CBPR approaches are built on these essential building blocks.

CBPR was used successfully with TGW in other settings. Furman et al. [29] provide examples of how participatory research with transgender communities supported a more remarkable social change. Additionally, studies have successfully drawn from CBPR approaches to shift the role of transgender people from research subjects to active participants [30, 31]. In addition to developing a checklist to critique positivist research methods and to guide researchers to adopt a CBPR approach, Singh et al. [30] highlight the importance of creating authentic relationships with transgender communities, collaborating with participants, and connecting research with advocacy.

One of the long-standing and successful examples of an epidemiological study using CBPR is that of the Transgender Community Health Project in San Francisco Bay, San Francisco, the United States of America [32]. The Transgender Community Health Project was established in 1996 in response to the concerns raised by the community regarding the little epidemiological data to document the health of transgender people and the lack of information on HIV infection amongst TGW. Ultimately, the Transgender Community Health Project was established in response to the lack of data and resulted in a partnership between the San Francisco Department of Public Health researchers and the transgender community [32]. According to Clements-Nolle and Bachrach [32], the key to the success of the Transgender Community Health Project is that it sprang from community concerns and priorities, most notably the lack of HIV epidemiological data for transgender people. This paper describes the CBPR approach used in the first bio-behavioral survey (BBS) to estimate HIV prevalence amongst TGW in South Africa.

### Description of the first bio-behavioral survey to estimate HIV prevalence amongst transgender women in South Africa

We conducted the BBS in three South African metropolitan areas: Buffalo City, Cape Town, and Johannesburg. Study sites were selected because of TGW civil society organizations (CSOs) in these areas. The researchers relied on CSOs that provide HIV prevention, treatment, and psychosocial support services to TGW in the South African metropolitan areas listed above to gain access to the community. For example, in 2010, the Social, Health, and Empowerment Feminist Collective of TGW in Africa (S.H.E.) was established in Buffalo City. S.H.E. supports the health, well-being, and human rights of TGW in Buffalo City. Gender DynamiX (GDX) and the Sex Workers' Advocacy and Education Taskforce (SWEAT) are based in Cape Town and provide advocacy, legal, and social support services for TGW. Access Chapter 2 (AC2), based in the Johannesburg metro-municipality, is a relative newcomer, established in 2016, and focuses on the health and human rights of transgender people.

We conducted the BBS in three phases. The first phase consisted of a rapid qualitative study. The overall aim of the rapid qualitative study was to establish a deeper and more nuanced understanding of the social and personal context that frames HIV risk for TGW, as well as the broader social settings and structures that place TGW at high risk for HIV infection. Following the rapid qualitative study, we implemented a pre-surveillance formative assessment. We conducted the rapid qualitative and pre-surveillance formative assessment between August 2017 to January 2018. Two objectives guided the pre-surveillance formative assessment: i) To assess the acceptability of recruiting TGW using respondent-driven sampling (RDS); ii) To identify the logistics needed to successfully implement HIV bio-behavioral surveys using RDS amongst TGW in the three metropolitan areas.

The BBS used RDS to recruit TGW from July 2018 to March 2019 in the South African metro municipalities of Buffalo City, Cape Town, and Johannesburg [24]. RDS has become a feasible option for a rigorous sampling of 'hard-to-reach' populations [33, 34]. RDS is a variant of chain-referral methodology that assumes that those best able to access members of a 'hard-to-reach' population are their peers [33, 35]. TGW form close ties and relationships with each other to build community resilience and often function as alternative kinship structures for those disowned by their families. A study conducted by Hwahng et al. [36] with TGW in New York City demonstrated that surrogate family relationships built community resilience and, in turn, individual strength [36]. The formation of close ties and relationships with other TGW had significant implications for the participation of TGW in our study, and we selected RDS as the most appropriate method for reaching TGW.

Using dried blood spots (DBS) and surveys, we collected data on HIV risk behaviors and linkage to care. We also performed HIV, antiretroviral (ARV), and viral load testing amongst TGW 18 years and older. In total, 887 transgender women (i.e., Buffalo City: n = 305; Cape Town: n = 259 and Johannesburg: n = 323) were recruited [24].

### Description of the CBPR approach used in the study

**Fig. 1:**
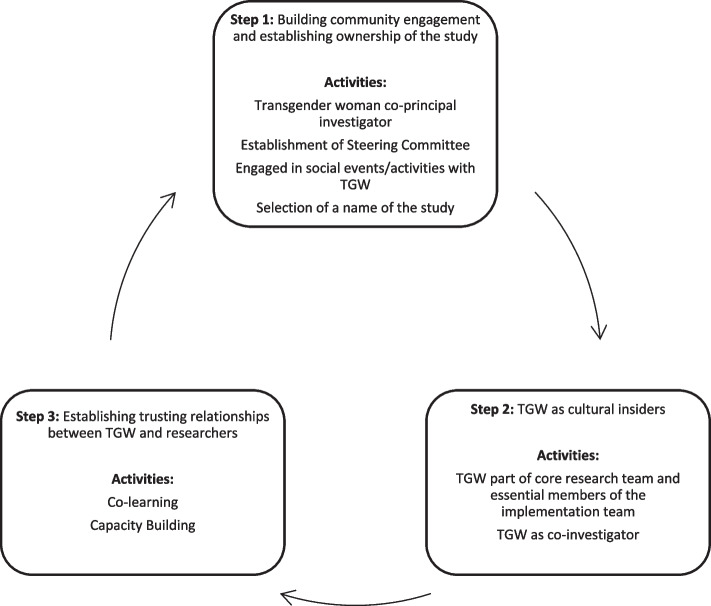
CBPR approach used in the *Botshelo Ba* Trans Study. This figure presents a description of the CBPR approach used in the *Botshelo Ba Trans* Study. Even though the CBPR approach used in this study is represented in a sequence of steps, the basic cycle emphasizes a circular flow, rather than a sequence of steps following on from each other. Step 1 entailed building community engagement and establishing ownership of the study. Activities that inform this step included the following: establishment of a Steering Committee; engaging in social events/activities with transgender women to engage with members of the community; whilst the third activity which was instrumental to establishing ownership of the study was the selection of a name for the study. Step 2 involved engaging with transgender women as cultural insiders. Specific activities that informed Step 2 included having TGW as part of the core research team and essential members of the implementation team and importantly having a TGW as a co-investigator on the study. Step 3 entailed establishing trusting relationships between TGW and researchers. Part of this step involved co-learning and capacity building

The researchers envisioned the BBS as a case study informed by a social justice paradigm. We applied this paradigm to all study phases, from project conceptualization, implementation, data analysis, and dissemination of findings. All these activities were underpinned by meaningfully involving TGW every step of the way. The envisioned model upon which the BBS was premised aimed to challenge the dominant research discourse: *on* communities rather than *with* or led by communities. This model challenges the power dynamics between the researcher and research participant (See Fig. 1).

#### Step 1: Building community engagement and establishing ownership of the study

The Steering Committee had to represent TGW in South Africa to reflect on the principles of using a CBPR-informed approach. For instance, although we conducted the study in urban metro-municipalities, TGW from small-town South Africa, including a township called *Botshabelo* in the Free State and the Matzikama sub-district, located about 300 km from the City of Cape Town, were invited to participate in the committee. Members of the research team approached TGW residents in the three South African metropolitan areas who were "influencers" and activists in the transgender movement. Most TGW had established trans-inclusive NGOs (i.e., representatives of S.H.E; GDX; SWEAT; AC2) in the selected metropolitan areas. Other key stakeholders invited to the Steering Committee included US Centers for Disease Control and Prevention (CDC) staff and technical advisors from the University of San Francisco, California. Steering Committee members conceptualized critical elements of the research study: advising on recruiting TGW, selecting the sampling methodology, defining the eligibility criteria, selecting initial study participants (i.e., seeds who initiate recruitment), and finalizing the survey instrument. Steering Committee members were actively disseminating study findings to the TGW community. We held Steering Committee meetings before implementing each study phase to ensure continuous consultation.

Secondly, our research team engaged in several activities that fostered trust and collaboration between the researchers and the networks of TGW in the three metropolitan areas. Members of the research team were involved in several activities organized by S.H.E. and GDX. Being visible at trans-specific events strengthened rapport. For example, the research team participated in a rally organized by GDX at the Cape Town High Court in support of Jade September, a transgender female prisoner. Jade September has turned to the High Court to compel the Departments of Justice and Correctional Services to allow her to dress as a woman, even though she is in a male prison. In Buffalo City, the research staff attended the annual Miss Trans Diva beauty pageant competition. The research team participated in the annual Queer Pride event in Cape Town. Participation effectively increased the study's visibility and demonstrated the researchers' commitment to the transgender community.

Our contact was not only limited to formal events. Researchers also "hung out" in clubs and other informal engagements. Establishing community engagement with TGW outside traditional institutions was strengthened using social media platforms such as Facebook and WhatsApp. The study had a dedicated Facebook page administered by a TGW. In addition to TGW-researcher communication, TGW were able to engage with other TGW and raise questions that they might have concerning the study.

Lastly, we engaged TGW in selecting a name for the study. We hoped that engaging TGW in choosing a name for the survey would foster ownership of the study amongst TGW. Members of the research team held informal group discussions with established support groups for TGW in the three metropolitan areas. We presented the information collected to our Steering Committee. The name chosen by members of the Steering Committee was the *Botshelo Ba Trans* Study. *Botshelo* (from Sesotho) means life, love, and happiness. We chose this name to celebrate the spirit of TGW, who are strong and brave in the face of constant societal discrimination [37].

#### Step 2: Transgender women as cultural insiders

Having an 'insider' perspective facilitated the recruitment of TGW. Notably, one of the study co-investigators identified as a TGW; this had significant implications in establishing ownership of the study and mobilizing TGW to participate. All screeners employed in the study were TGW; they were the first point of contact for potential participants. Per the study protocol, a screener's role was to verify study eligibility, explain study procedures, and assist with participant intake. At least one interviewer at all research sites was a TGW. Screeners and interviewers were well respected amongst their peers and were considered veterans of the transgender movement. They harnessed their connections with other TGW through their networks and mobilized young TGW and new advocates to participate.

#### Step 3: Establishing trusting relationships between transgender women and researchers

Researchers were also mindful of the historical context of TGW's mistrust and suspicion of the research sector and other formal institutions. Research staff created a pleasant on-site atmosphere and occasionally provided refreshments for participating TGW. A friendly on-site atmosphere and the occasionally provisioned refreshments helped ease participant frustration, especially on busy days. Provisional refreshments, however, established an expectation from recruits and had the negative effect of creating duplicate recruits (i.e., participants taking part in the study twice) and recruits bringing others who were not eligible (i.e., cisgender women). On a positive note, we found that TGW often visited the site after survey participation to collect their secondary incentives and "hang out."

Moreover, TGW often accessed the three research sites requesting employment opportunities and support in accessing health- and gender-affirming care. The sites evolved into "safe spaces" for TGW. Using the offices as "safe spaces" required monitoring as, at times, activity levels of TGW threatened to disrupt the research process. Careful negotiation was needed to ensure that the research was not disrupted.

Capacity building was an essential factor in creating trust between researchers and TGW. Researchers provided training on research ethics, basic interviewing skills, and RDS methodology—an accredited laboratory conducted specialized training for DBS and whole blood collection. Importantly, capacity building and training were collaborative. We completed the RDS staff training in a process where "the experiential knowledge [of TGW] was integrated into scientific knowledge" [38].

In this space, TGW were able to educate researchers on issues that are important to their health and well-being. Researchers and TGW were involved in co-learning, providing researchers with an improved understanding and empowering TGW.

In total, we trained 18 project staff members in RDS. Six were post-graduate-level educated project staff members in the social sciences. One of the six was also qualified as a professional nurse. The six post-graduate-level educated project staff members had prior research experience as post-graduate students or research assistants at university or NGO-based research projects. Three were registered professional nurses employed in the study in this position. The remaining nine had all completed their high school education. Most of the TGW employed as staff members had excellent activist, organizational, and networking skills than prior research experience.

Twenty-four participants attended a weeklong scientific writing workshop. The 24 participants comprised of members of the Steering Committee, research participants, and project staff members. The writing workshop was primarily a capacity-building exercise and elicited experiential knowledge of TGW. This workshop also provided TGW with the space to engage with the processes of scientific writing and contribute to validating the study findings. The engagement of research participants in scientific writing challenges the status quo where researchers often infer knowledge onto people (i.e., research extraction) to a collaborative process that is gender-sensitive and community-led. The engagement of research participants in scientific writing is the meaningful engagement called for in the multi-agency *TRANSIT* tool developed to implement HIV and STI services with transgender communities [39].

Study staff conducted ongoing informal participant observation during the study's implementation in the research sites' waiting rooms. In addition to participant observation, study staff also engaged in informal discussions with study participants who completed the first and second visits. Continuous feedback on any challenges experienced by the community was raised during these discussions, and remedial actions were taken. In addition, these informal discussions also strengthen the trust established early in the study. One issue that arose and was addressed was that the travel reimbursement was insufficient compensation. The travel reimbursement was increased; we could reach participants further away. However, following the increase in travel reimbursement, there was again an increase in ineligible participants. An increase in ineligible participants may have also been due to network saturation; in response, screeners were re-trained to probe and have long discussions with potential participants regarding their self-identification and eligibility criteria.

## Discussion

There are several advantages to using CBPR. Guta, Flicker, and Roche [40] list the benefits of participatory research as two-fold: firstly, the ability to collect data that reflects the community and its needs, and secondly, it provides the prospect to capacitate and upskill individuals in the community. CBPR emphasizes the strengths and resources of every partner by valuing co-research, empowerment, and capacity building, combining knowledge and bi-directional leadership [41]. This approach contrasts sharply with traditional research, in which the academic is the expert who conducts research, with little or no input from the participants or community under study [42]. Building connections and establishing ownership of the survey evoked a meaningful engagement with the study that might not have been developed within traditional research paradigms. By following a CBPR-informed approach in the *Botshelo Ba Trans* study, grassroots TGW were involved in conceptualizing, implementing, and analyzing the data. Such collaboration enhanced the validity and usefulness of study findings and created opportunities for taking the data beyond the research study. The process of co-creation/co-learning was an advantage to both researchers and TGW in the *Botshelo Ba Trans* study. In the study, multiple perspectives from the research community and the TGW community were included that contributed to a more nuanced and richer interpretation and analysis of the data. Not only were TGW involved in a way that provided them with the skills and capacity to conduct research, but researchers also increased their own knowledge of sexual orientation and gender identity as it relates to TGW, such as understandings of the language and norms of the community and their life experience. By making use of a CBPR approach, the *Botshelo Ba Trans* study changed the status of the community of TGW to partners in the research process [43]. TGW reported a meaningful participation in the study and a capacity to influence the implementation of the study. This generated an investment in the study, which was of vital importance when difficulties were encountered. For instance, when difficulties were encountered to reach the required sample size, members of the transgender community in the Steering Committee and the study staff contributed with innovative ideas and additional effort to ensure that targets were met.

An insider perspective proved essential to the study's overall success. Like the stakeholders of the *Botshelo Ba Trans* study, female sex worker (FSW) stakeholders in an FSW BBS highlighted the importance of incentives and selection in the location of RDS sites [44]. Both these methodological concerns were essential issues raised by TGW stakeholders as well. Both groups indicated that the RDS site should be located in a familiar and easily accessible area, discreet from the public eye [44]. Given that this study also included the study funders, the CDC, as co-investigators, extended the insider views and provided TGW affiliated with CSOs a more meaningful sense of control and influence within the operations of the study.

In establishing trusting relationships between TGW and researchers, research staff created a pleasant on-site atmosphere and occasionally provided refreshments and beverages for TGW. A friendly on-site atmosphere and the occasional provision of refreshments contributed to establishing trusting relationships between TGW and researchers; however, it also increased the number of ineligible participants visiting the site (i.e., cisgender women). Other complications included staff safety and office space: often, the BBS led to overcrowding and boisterous behavior at the interview sites, which interfered with study operations. Management of rowdy behavior or restricting the use of interview sites as safe spaces also generated uneasiness between study staff and participants. The extraordinary result of the research sites as safe spaces for TGW is perhaps a reflection of the lack of safety in the world of TGW.

The attitude of the research team in engaging with the community must be authentic and not seen as using CBPR as purely a strategic initiative. The target community will quickly notice strategic and disingenuous relationships, especially those familiar with social rejection and stigmatization. In this study, the research team attended popular social events such as beauty pageant competitions and engaged positively with TGW. Researchers who engaged with TGW at social events allowed for interactions between researchers and TGW to become more relaxed and open.

This study has several limitations. Using a CBPR-informed approach increased the likelihood of the study being inclusive of the community's needs and TGW in leadership roles and those at grassroots levels. Likely, TGW from specific sectors of the community were still excluded. The focus falling on the CSOs meant those connected with these CSOs were more likely to be included. Those TGW still struggling to come to terms with their identity and those too anxious to make their trans identification public may still have been excluded. There are few realistic options for reaching this more hidden population.

The politicized nature of the study, by taking a CBPR and social justice approach, may have influenced how study participants participated and the responses they provided. Taking a CBPR and social justice approach may have prevented more socially and politically conservative TGW from participating.

Researchers defined transgender as someone whose gender identity does not conform to their gender at birth. However, this definition of being TGW may have excluded some participants who identify as women but have not had gender-affirming surgery or hormonal interventions. In addition, as stated above, some cisgender women arrived to participate in the study.

### Lessons learned

While implementing the *Botshelo Ba Trans* study, we learned lessons that might be useful for future surveillance work conducted with TGW in South Africa.

First, reaching and maintaining good relationships with transgender activists and leaders was integral to the study's success. Maintaining good relationships with these gatekeepers was formally constituted by establishing the Steering Committee. Thus, a fully representative Steering Committee was the first step in building rapport and connecting with TGW. Hence, we identified the gatekeepers early in this study and built on their strengths, resources, and relationships with TGW across the three metropolitan areas. According to Andrews et al. [45] CBPR approaches depend on existing strengths and resources instead of reinventing new structures and processes. Through engagement with the leaders of the transgender movement in South Africa, we ensured that the study was conducted within a social justice paradigm. In this way, the research prioritized the primary healthcare and social needs of TGW. The Steering Committee members made meaningful decisions during the study.

Secondly, TGW taking ownership of the study was vital to the study's success; the methodology described above allowed for a shift in power from the researchers to TGW. Taking ownership of the study is essential in TGW identifying with the survey, claiming it as their own, and contributing towards engendering a positive relationship with participants entering the study as they felt comfortable. The TGW employed as study staff demonstrated ownership of the survey by challenging decisions made by researchers and contributing their ideas, thereby contributing to better overall solutions.

Thirdly, researchers transferred research skills to TGW, further empowering this population. Knowledge production was not a one-way process but collaborative, with a dedicated sharing of lay and scientific knowledge. Thus as Clements-Nolle and Bachrach [32] note in the description of the Transgender Community Health Project based in San Francisco: CBPR is a co-learning process where there is a reciprocal transfer of knowledge. In the latter project, transgender community members, in partnership with research associates, spent many hours educating the health department researchers about issues specific to the transgender community, including the importance of self-identity and psychosocial reasons for risk-taking [32]. Finally, TGW often visited our research sites for information and advice on legal matters, social support, or gender-affirming healthcare. In reaching TGW in future studies, methods should consider complementary services that would benefit the TGW community. These services could include referral to gender-affirming care, legal gender recognition, and other services. Services like these are usually provided through service providers or intervention development research, however descriptive studies such as this have a role in linking participants in need to services and in contributing to the pressure on existing service providers to extend relevant services to TGW.

## Conclusion

A CBPR-informed approach was used in this study to allow the research team to address the context and needs of TGW meaningfully. While the aims of the study were set in advance, there was space to adapt these and to develop the methodological approach in conjunction with representatives of the transgender community. The methodological approach used in this study was facilitated by the Steering Committee, including members from the transgender community, researchers, funders, and other key stakeholders in conjunction with representatives of the transgender community. The use of CBPR facilitated the deepening of access, the recruitment of participants, and the participants' openness to the study, which arguably would improve honesty, the interpretation of data, and, finally, the validity of the data. TGW who participated also benefited by learning about research and other skills attached to the research process, increasing their commitment to the study. Based on our experiences, a CBPR-informed approach should be used in all studies with members of hard-to-reach populations. While the potential for this may vary, and indeed, the workload is increased, the full participation of the target population will contribute significantly to the implementation process and the results obtained.

## Data Availability

Data sharing is not applicable to this article.

## Abbreviations

AC2: Access Chapter 2
ARV: Antiretroviral
BBS: Bio-behavioral survey
CBPR: Community-based participatory research
CDC: US Centers for Disease Control and Prevention
CSOs: Civil society organizations
DBS: Dried blood spots
FSW: Female sex worker
GDX: Gender DynamiX
HIV: Human Immunodeficiency Virus
HSRC: Human Sciences Research Council
NGO: Non-governmental organization
RDS: Respondent-driven sampling
REC: Research Ethics Committee
S.H.E.: Social, Health, and Empowerment Feminist Collective of Transgender Women in Africa
STI: Sexually transmitted infection
SWEAT: the Sex Workers’ Advocacy and Education Taskforce
TGW: Transgender women
